# Acute Liver Failure After Administration of Acetaminophen at the Recommended Daily Dose in an Adult: A Case Report

**DOI:** 10.7759/cureus.45735

**Published:** 2023-09-21

**Authors:** Masafumi Fukuda, Nobuhisa Hirayu, Masakazu Nabeta, Osamu Takasu

**Affiliations:** 1 Intensive Care Unit, Advanced Emergency and Critical Care Center, Kurume University Hospital, Kurume, JPN; 2 Department of Emergency and Critical Care Medicine, Kurume University School of Medicine, Kurume, JPN

**Keywords:** side effects, n-acetylcysteine, body weight, acetaminophen, acute liver failure

## Abstract

Acetaminophen may cause liver damage in a dose-dependent way: we experienced a case where an intravenous injection of 3 g/day of acetaminophen, which is less than the recommended maximum dose, was thought to have caused acute liver failure in a 73-year-old female. Four courses of postoperative adjuvant chemotherapy were given, without liver damage until the third course. After the administration of the fourth course, the patient experienced nausea and vomiting. She was admitted to the hospital with a diagnosis of enteritis a week later. At the time of admission, there was no liver impairment. For abdominal pain caused by enteritis, acetaminophen was administered intravenously over two days, totaling 4,000 mg. On the third day, acute liver failure developed, and N-acetylcysteine was administered. There was no improvement after the introduction of treatment; hence, 1,000 mg/day of steroid pulse therapy was administered. The patient’s liver function started to improve, and she was discharged from the hospital two weeks later. This case suggests that the amount of acetaminophen used per unit of body weight may be unintentionally greater for adults with a small physique; thus, physicians should provide sufficient monitoring to discover side effects early and ensure there is appropriate use.

## Introduction

Liver damage is a problematic side effect of acetaminophen, with liver toxicity generally considered to be dose-dependent [1]. The current maximum daily recommended dose of acetaminophen is 4 g/day for adults [2], but on this occasion, we experienced a case in which 3 g/day of acetaminophen, which is lower than the recommended maximum dose, is suspected to have caused acute liver failure. The patient consented to using her clinical data in a published scientific report.

## Case presentation

Our patient was a 73-year-old female with a history of hypertension and dyslipidemia, who was being treated with 5 mg/day of amlodipine for hypertension and 2 mg/day of pitavastatin for dyslipidemia. She had no history of hepatitis and no family history of liver damage. She was not a regular drinker. Postoperative adjuvant chemotherapy (docetaxel + cyclophosphamide) was being given for right breast cancer, with no episodes of drug-induced liver injury until the third course. After the fourth course of docetaxel + cyclophosphamide, the patient had a lack of appetite, nausea, and vomiting. A week after administration of the fourth course, she became unable to consume food orally and was admitted to her regular hospital. Based on the medical findings and pain in the epigastric region, she was deemed to have enteritis associated with chemotherapy. In the blood tests upon admission, there was no evidence of liver impairment. We confirmed that the patient had not taken acetaminophen orally before admission. Therefore, for pain relief, 1,000 mg of acetaminophen was administered intravenously on the same day, followed by an intravenous infusion of 3,000 mg the following day. Blood tests on the third day after the first administration of acetaminophen showed an increase in aspartate transaminase (AST) to 2,001 U/L, an increase in alanine aminotransferase (ALT) to 1,255 U/L, and a reduction of prothrombin time (PT) down to 30%. Abdominal contrast-enhanced computed tomography showed homogeneous liver enhancement without thrombosis, and there were no signs of significant liver enlargement or atrophy. Acetaminophen could not be ruled out as the cause of liver failure; hence, on the fourth day after the initial administration of acetaminophen, N-acetylcysteine was administered at a dose of 140 mg/kg (5.6 g). Subsequently, N-acetylcysteine was administered every four hours at a dose of 70 mg/kg (2.8 g). Liver function further deteriorated in the blood test on day 5 after the initial administration of acetaminophen, and the patient was admitted to the intensive care unit (ICU) for intensive treatment of acute liver failure once values of AST increased to 3,676 U/L, ALT increased to 4,086 U/L, and PT decreased to 26%. Her body weight at the time of hospital admission was 43.8 kg, and her body mass index was 20.5 kg/m^2^. At the time of transfer to the ICU, the patient’s temperature was 37.9 ℃, pulse was 110 beats/minute, respiration was 27 breaths/minute, and blood pressure was 132/62 mmHg. After surgery on the right side of the chest, the physical findings were only mild yellowing of the bulbar conjunctiva, with no other significant findings and no flapping tremors. The blood test results at the time of transfer are shown in Table 1.

**Table 1:** Laboratory data upon ICU admission. pH, power of hydrogen; PaO_2_, partial pressure of arterial oxygen; PaCO_2_, partial pressure of arterial carbon dioxide; HCO_3_, hydrogen carbonate; BE, base excess; WBC, white blood cell; Hb, hemoglobin; Ht, hematocrit; Plt, platelet; PT, prothrombin time; APTT, activated partial thromboplastin time; FDP, fibrin degradation product; AFP, alpha fetoprotein; PIVKAⅡ, protein induced by vitamin K absence or antagonist II; AST, aspartate transaminase; ALT, alanine transaminase; LDH, lactate dehydrogenase; γ-GT, γ-glutamyl transpeptidase; ChE, cholinesterase; BUN, blood urea nitrogen; Cre, creatinine; CRP, C-reactive protein; ANA, anti-nuclear antibody; COI, cutoff index; HAV, hepatitis A virus; HCV, hepatitis C virus; EBV, Epstein-Barr virus; CMV, cytomegalovirus; HSV, herpes simplex virus; Ig, immunoglobulin

Biochemical analysis		
	Results	Reference range
Blood gas analysis		
pH	7.460	7.380–7.460
PaO_2_	182 mmHg	74.0–109.0 mmHg
PaCO_2_	34.3 mmHg	32.0–46.0 mmHg
HCO_3_	24.3 mEq/L	21.0–29.0 mEq/L
BE	0.8 mEq/L	-2 to 2 mEq/L
Lactate	2.6 mmol/L	0.44–2.13 mmol/L
Hematology		
WBC	22.1 × 10^3^/µL	3.3× 10^3^ to 8.6 × 10^3^/µL
Hb	10.4 g/dL	11.6–14.8 g/dL
Ht	31.1%	35.1%–44.4%
Plt	41.0 × 10^4^/µL	15.8 × 10^4^ to 34.8 × 10^4^/µL
Coagulation		
PT	18%	80%–120%
APTT	43.2 s	24.0–39.0 s
Fibrinogen	241 mg/dL	200–400 mg/dL
FDP	25.7 µg/mL	<5 µg/mL
Antithrombin	46%	80%–130%
Ig		
IgA	200 mg/dL	93–393 mg/dL
IgM	63 mg/dL	45–300 mg/dL
IgG	825 mg/dL	800–1,750 mg/dL
Tumor markers		
AFP	27.7 ng/mL	<7 ng/mL
PIVKAⅡ	66 mAU/mL	<40 mAU/mL
Biochemistry		
AST	4295 U/L	13–30 U/L
ALT	4885 U/L	7–30 U/L
LDH	4,707 U/L	124–222 U/L
γ-GT	139 U/L	9–32 U/L
Total bilirubin	2.2 mg/dL	0.4–1.2 mg/dL
Direct bilirubin	1.1 mg/dL	<0.2 mg/dL
CHE	137 U/L	201–421 U/L
Total protein	5.6 g/dL	6.6–8.1 g/dL
Albumin	3.7 g/dL	4.1–5.1 g/dL
Total cholesterol	91 mg/dL	142–219 mg/dL
Ammonia	137 µg/dL	12–66 µg/dL
BUN	20 mg/dL	8–20 mg/dL
Cre	0.39 mg/dL	0.46–0.79 mg/dL
CRP	0.46 mg/dL	≤0.14 mg/dL
ANA	<40	<40
Viral markers		
IgM-HAV Ab	(–)	
HBs-Ag	0.001 IU/mL	<0.005 IU/mL
HBs-Ab	36.8 mIU/mL	<10 mIU/mL
HBc-Ab	0.1 COI	<1.0 COI
HCV-Ab	0.1 COI	<1.0 COI
IgM-EBV VCA Ab	<10	<10
IgG-EBV VCA Ab	80	<10
IgM-CMV Ab	1.87	<1.0
IgG-CMV Ab	8.2 AU/mL	<6.0 AU/mL
IgM-HSV Ab	0.41	<0.8
IgG-HSV Ab	36.9	<4.0

**Figure 1:**
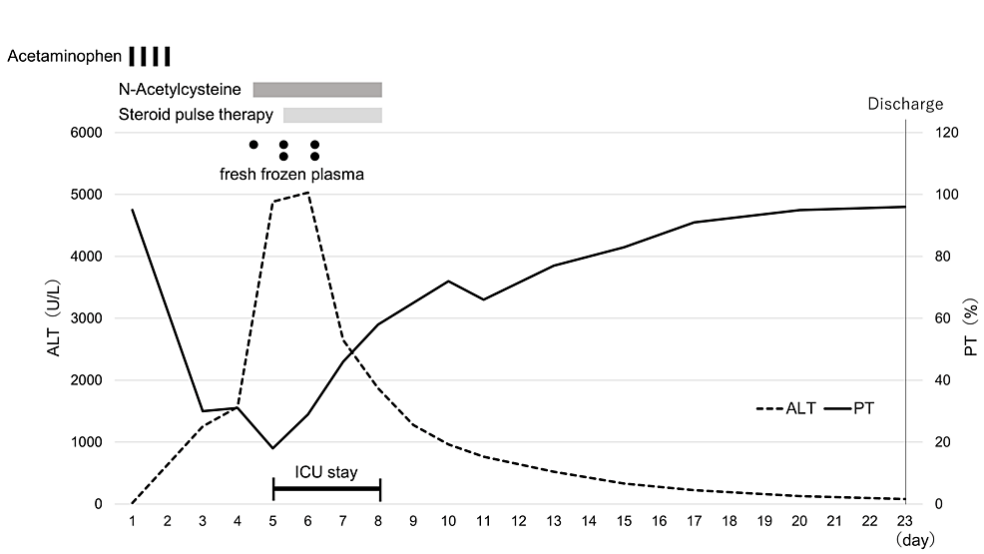
Clinical course of the present patient. A visual description of the patient's case. ALT and PT are displayed in this figure, with the top portion of the graph representing various interventions during hospitalization. ALT, alanine transaminase; ICU, intensive care unit; PT, prothrombin time

AST and ALT levels increased even more, and hyperammonemia and hyperbilirubinemia were additionally observed. No virus- or collagen disease-related test abnormalities that may cause liver function impairment were seen. From the history of disease and elimination of other causes, acute liver failure due to acetaminophen was diagnosed. In addition to the treatment given by the previous physician, the patient received 1,000 mg/ day of steroid pulse therapy and fresh frozen plasma (FFP) transfusion. The course of treatment is shown in Figure 1.

The ALT level increased further after two days in the ICU, but the AST level was reduced. The ALT level was reduced on the third day after entering the ICU; hence, administration of FFP, steroid pulse therapy, and N-acetylcysteine ended. There was progression without deterioration even after the end of treatment; therefore, the patient was released from the ICU on the fourth day and was transferred to her regular hospital on the 18th day.

## Discussion

Liver damage due to acetaminophen onsets in a dose-dependent manner; hence, administration at a dose less than the recommended 4 g/day is usually considered safe [3]. However, in this case, the patient suffered from acute liver failure onset despite the use of a dose lower than the recommended dosage. Liver impairment due to acetaminophen is known to derive from its active metabolite, N-acetyl-p-benzoquinone-imine (NAPQI) [4]. Although acetaminophen undergoes sulfate conjugation and glucuronidation in the liver, some acetaminophen is converted to NAPQI by cytochrome P450 during the administration of an overdose. NAPQI is detoxified by glutathione conjugation, but depletion of glutathione increases NAPQI and causes liver damage [5]. In this case, we suspect that patient background factors contributed to liver failure, as undernutrition, drinking, and a history of liver damage are known risk factors for liver damage caused by acetaminophen [6]. In this case, although there was no malnutrition, the patient’s oral food intake decreased for about a week due to the effect of enteritis associated with postoperative chemotherapy, and hypoalbuminemia and hypocholesterolemia blood tests indicated that the patient was undernourished. In addition, the fact that the patient was of a small frame is thought to have impacted the onset of liver damage. Guidance for intravenous administration of acetaminophen in the United Kingdom, United States, and Australia recommends 60 mg/kg/day as the maximum dose for adults weighing less than 50 kg due to concerns over liver damage, although this recommendation is not based on high-quality clinical data [7]. In this case, the maximum daily dose of 4,000 mg/day was strictly adhered to, but the body weight equivalent was 68.5 mg/kg/day, which unintentionally exceeded the recommended dose according to these guidelines. Furthermore, the bioavailability of intravenous administration is higher than that of oral administration, which is between 0.7 and 0.9 [8], and may also have been a factor in triggering liver damage. Hence, particular care is required when administering acetaminophen intravenously.

## Conclusions

Acute liver failure due to an overdose of acetaminophen is often fatal. Given that even the recommended dose may result in an overdose, a dose reduction and the administration route should be considered and physicians must put effort into sufficient monitoring for and early discovery of side effects, particularly when using acetaminophen for malnourished or underweight adults.

## References

[1] Jaeschke H (2015). Acetaminophen: dose-dependent drug hepatotoxicity and acute liver failure in patients. Dig Dis.

[2] (2009). Food and Drug Administration, HHS. Organ-specific warnings; internal analgesic, antipyretic, and antirheumatic drug products for over-the-counter human use; final monograph. Final rule. Fed Regist.

[3] Watkins PB, Kaplowitz N, Slattery JT, Colonese CR, Colucci SV, Stewart PW, Harris SC (2006). Aminotransferase elevations in healthy adults receiving 4 grams of acetaminophen daily: a randomized controlled trial. JAMA.

[4] Jollow DJ, Thorgeirsson SS, Potter WZ, Hashimoto M, Mitchell JR (1974). Acetaminophen-induced hepatic necrosis. VI. Metabolic disposition of toxic and nontoxic doses of acetaminophen. Pharmacology.

[5] Heard KJ (2008). Acetylcysteine for acetaminophen poisoning. N Engl J Med.

[6] Lee WM (2004). Acetaminophen and the U.S. Acute Liver Failure Study Group: lowering the risks of hepatic failure. Hepatology.

[7] Caparrotta TM, Antoine DJ, Dear JW (2018). Are some people at increased risk of paracetamol-induced liver injury? A critical review of the literature. Eur J Clin Pharmacol.

[8] Forrest JA, Clements JA, Prescott LF (1982). Clinical pharmacokinetics of paracetamol. Clin Pharmacokinet.

